# Thermodynamic study of the adsorption of acridinium derivatives on the clay surface†

**DOI:** 10.1039/d0ra03158e

**Published:** 2020-06-04

**Authors:** Yuma Yoshida, Tetsuya Shimada, Tamao Ishida, Shinsuke Takagi

**Affiliations:** Department of Applied Chemistry for Environment, Graduate School of Urban Environmental Sciences, Tokyo Metropolitan University 1-1 Minami-ohsawa Hachioji-shi Tokyo 192-0397 Japan; Research Center for Gold Chemistry, Tokyo Metropolitan University 1-1 Minami-ohsawa Hachiohji-shi Tokyo 192-0397 Japan; Research Center for Hydrogen Energy-based Society (ReHES), Tokyo Metropolitan University 1-1 Minami-ohsawa Hachiohji-shi Tokyo 192-0397 Japan takagi-shinsuke@tmu.ac.jp +81 42 677 2839

## Abstract

In this study, the adsorption behavior of mono-cationic acridinium derivatives on a synthetic clay mineral (Sumecton SA) was investigated. The acridinium derivatives were adsorbed on the clay surface without aggregation, as found from the changes in the absorption spectra of the acridinium derivatives with SSA and without SSA represented by two-component equilibrium systems of adsorbed and non-adsorbed components. Following the Langmuir isotherm analysis, the adsorption equilibrium constants and maximum adsorption amounts were determined for acridinium derivatives, and the Gibbs free energy change (Δ*G*) was calculated to be in the range of −33.8 to 40.0 kJ mol^−1^ from the adsorption equilibrium constants. These results indicated that the adsorption of acridinium derivatives on the clay surface was an exergonic reaction. Moreover, thermodynamic parameters such as enthalpy change (Δ*H*) and entropy change (Δ*S*) were obtained from the temperature effect experiments. For all acridinium derivatives, Δ*H* (from −7.82 to −26.0 kJ mol^−1^) and Δ*S* (0.047–0.088 kJ mol^−1^ K^−1^) were found to be negative and positive, respectively. It was suggested that not only electrostatic interactions, but also van der Waals forces and hydrophobic interactions played an important role in the adsorption of cationic aromatic molecules on the clay surface. Because these thermodynamic parameters showed a strong correlation with the molecular cross-sectional area of acridinium derivatives, it was suggested that the contribution of hydrophobic interactions became smaller as the molecular cross-sectional area became larger.

## Abstract 2

Thermodynamic studies indicate that van der Waals and hydrophobic interactions contribute to the adsorption of mono-cationic acridinium derivatives on the clay surface.

## Graphical abstract

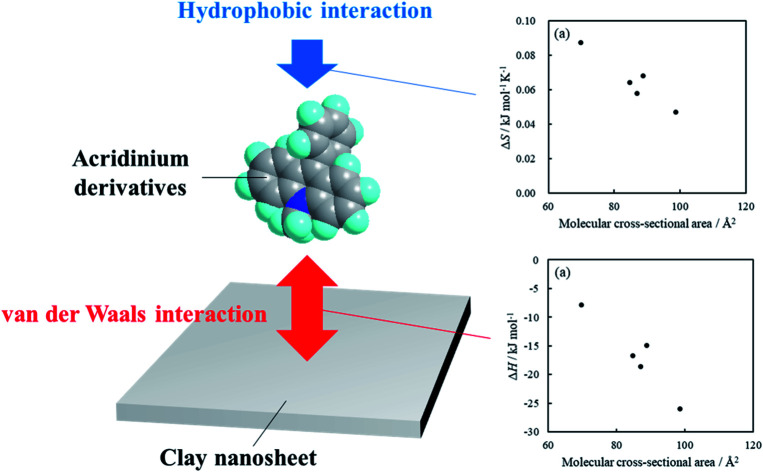

## 1. Introduction

**Fig. 1:**
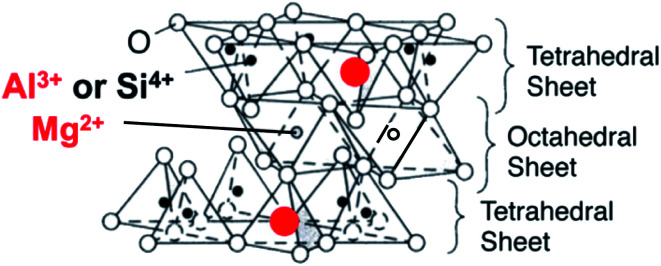
The unit structure of saponite.

The adsorption behavior of organic compounds on a solid surface is worth investigating from the viewpoints of basic and applied science. However, the adsorption behavior is complicated in general mainly because of the non-uniform solid surface. It is known that clay minerals provide an ideal solid surface, which is flat at the atomic level. Therefore, we chose clay minerals as a substrate to investigate the adsorption behavior of organic compounds. Saponite, which has two tetrahedral and octahedral layers, is a typical layered material. The isomorphic substitution of Si^4+^ by Al^3+^ in the tetrahedral layer produces negative charges on the surface of the clay minerals (Fig. 1).

By utilizing these negative charges on the clay surface, cationic species can be easily adsorbed on the clay surface.^1–4^ Several researchers have widely investigated the use of clay minerals as a host material for photo-functional materials and as adsorbents.^1–9^ To date, various studies have been conducted on the adsorption behavior of cationic molecules on the clay minerals. However, cationic molecules tend to aggregate on the solid surface due to the interaction between guest molecules, and this makes the analysis of the adsorption behavior difficult.^10–12^ On the other hand, we found that the specific cationic molecules such as cationic porphyrins are adsorbed on the clay surface without aggregation even at high density.^13–15^ The suppression of the aggregation of guest molecules makes the adsorption and photochemical behavior of the adsorbed molecules simple. The intrinsic photochemical properties of the adsorbed dyes can be observed under such conditions. For example, we found that the emission from guest molecules was significantly enhanced by the adsorption on the clay surface.^16–19^ This effect is called “Surface-Fixation Induced Emission (S-FIE)”. Concerning the adsorption behavior on nanosheet surfaces, to date, the published research studies^20–23^ are ambiguous because most systems include interactions between the adsorbate and adsorbate, which is aggregation, in addition to those between the adsorbent and the adsorbate.

**Fig. 2:**
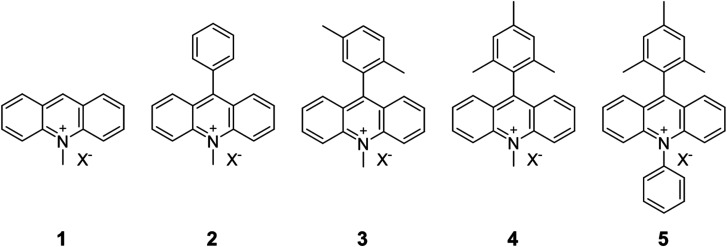
Structures of acridinium derivatives. The counterion X^−^ is perchlorate or chloride.

In this study, we investigated the adsorption behavior of acridinium derivatives as cationic organic guest molecules on saponite. By choosing suitable adsorbates and conditions in which the nanosheets were exfoliated, the interaction between the adsorbent and the adsorbate without aggregation could be clearly discussed. The molecular structures of the acridinium derivatives are shown in Fig. 2. Acridinium derivatives, such as 9-mesityl-10-methylacridinium, can generate a long-lived electron-transfer state due to their orthogonal geometry and have attracted significant attention as photo-redox catalysts.^24–26^ Moreover, extensive efforts have been devoted to improving their photo-function and developing solid catalysts by incorporating acridinium derivatives into the adsorbent.^27–29^ On the other hand, little is known on the adsorption of acridinium derivatives on the clay surface.^30,31^ In this research, the adsorption behavior of acridinium derivatives was investigated by comparing the thermodynamic parameters for adsorption.^32,33^ This study is expected to clarify the effect of the molecular structure on the adsorption of cationic molecules on the clay surface and which interactions are dominant.

## 2. Experimental section

### 2.1. Materials

Clay minerals (saponite): Sumecton SA (SSA, [(Si_7.2_Al_0.8_)(Mg_5.97_Al_0.03_)O_20_(OH)_4_]^−0.77^(Na_0.49_Mg_0.14_)^+0.77^) as a synthetic saponite was purchased from Kunimine Industries Co., Ltd. and used without further purification. The unit structure is shown in Fig. 1. SSA was analyzed with AFM, XRD, XRF and FT-IR spectroscopy, as described in a previous paper.^13^ Judging from AFM measurements, the shape of SSA was disc-like and the diameter was 20–50 nm. The specific surface area and the cation exchange capacity (CEC) of SSA were 750 m^2^ g^−1^ and 9.97 × 10^−4^ equiv. g^−1^, respectively.^34^ The area occupied per one negative charge calculated from the specific surface area and CEC was 1.25 nm^2^. The aqueous dispersion of saponite nanosheets whose particle size was small (<100 nm) was substantially transparent in the UV-visible range. Water was deionized with an ORGANO BB-5A system (PF filter × 2 + G-10 column). 10-Methyl-9-phenylacridinium perchlorate, 9-(2,5-dimethylphenyl)-10-methylacridinium perchlorate and 9-mesityl-10-methylacridinium perchlorate were purchased from Tokyo Kasei (Japan). 10-Methylacridinium methyl sulfate and 9-mesityl-10-phenylacridinium tetrafluoroborate were purchased from Aldrich. The counterion was changed to perchlorate or chloride with an ion-exchange resin (Organo, Amberlite Resin IRA-400 treated with HClO_4_ or HCl).

### 2.2. Analysis

TG-DTA curves were measured with a Shimadzu DTG-60H analyzer to determine the water content of acridinium derivatives and SSA. Absorption spectra were obtained on a UV-3150 UV-vis spectrophotometer (SHIMADZU). Molecular models of acridinium derivatives were depicted by a molecular drawing software (*i.e.*, Chem 3D). The structures of the molecular models were optimized by the semiempirical molecular orbital method using MOPAC 2016. Calculations were carried out by using the following command: PM6 CHARGE = 1 GRAPHF AUX BONDS DENSITY PI ENPART MMOK. The height of acridinium derivatives from their aromatic plane was calculated by the optimized structure. The projected cross-section of acridinium derivatives horizontal to the aromatic plane was defined as the molecular cross-section and was calculated by using an image processing software (Image J). Charge density at nitrogen atoms and energy levels of HOMO and LUMO in acridinium derivatives were calculated by DFT calculations performed at the B3LYP/6-31G* level using Gaussian09 package.^35^

### 2.3. Sample preparation for the acridinium derivatives/clay complex

First, 1.0 × 10^−4^ M acridinium derivative stock solutions were prepared in water; 1.0 × 10^−4^ equiv. L^−1^ SSA stock dispersion was prepared in water. To prepare the acridinium derivative–clay complex, the acridinium derivative aqueous solution and SSA aqueous solution were mixed at an arbitrary rate and diluted with water under stirring in a quartz cell (1.0 × 1.0 cm). The concentration of acridinium derivatives was adjusted in the concentration range of 2.28 × 10^−6^ to 2.59 × 10^−5^ M. The concentration of SSA dispersions was adjusted in a concentration range of 1.06 × 10^−5^ to 1.40 × 10^−5^ equiv. L^−1^. By changing the volume of the acridinium derivative stock solution and SSA dispersion, the adsorption density of the acridinium derivatives was adjusted to be 16.3, 24.5, 32.6, 40.8, 48.9, 57.1, 65.2, 73.4, 81.5, 97.8, 122.3, 163.0, 244.6% *vs.* CEC of SSA.

## 3. Results and discussion

### 3.1. Adsorption behavior of acridinium derivatives on SSA

**Fig. 3:**
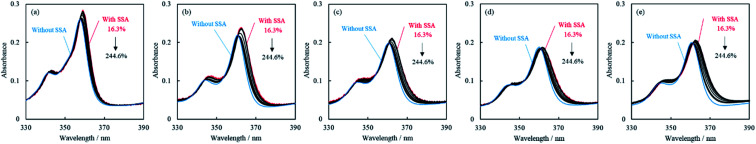
UV-vis absorption spectra of acridinium derivatives with SSA and without SSA in water at 298.15 K. The loading level of each acridinium derivative was 16.3, 24.5, 32.6, 40.8 48.9, 65.2, 81.5, 122.3, 163.0, 244.6% *vs.* CEC: (a) compound 1, (b) compound 2, (c) compound 3, (d) compound 4, (e) compound 5. The spectra were corrected with each acridinium derivative concentration.

The adsorption behavior was examined by measuring the UV-vis absorption spectra of the acridinium derivatives. The UV-vis absorption spectra of the acridinium derivatives with SSA and without SSA in water are shown in Fig. 3. The spectra of the acridinium derivatives with SSA at 16.3% *vs.* CEC showed a redshift of approximately 3 nm compared to the spectra of the acridinium derivatives without SSA.

The redshift in absorption maximum indicated the adsorption of the acridinium derivatives on the clay surface. It is known that the causes of this phenomenon are molecular structural changes^5,13,36,37^ or the formation of aggregates on the clay surface.^38,39^ The absorption spectra of acridinium derivatives with SSA and without SSA showed isosbestic points with the increase in the loading level. This result indicated that these spectral changes were represented by two-component equilibrium systems of adsorbed and non-adsorbed components. If aggregates are formed, the isosbestic point cannot be observed. Therefore, it was suggested that the spectral change in the acridinium derivatives on the clay surface was not because of the formation of aggregates but because of molecular structural changes.

### 3.2. Langmuir adsorption isotherms

An adsorption isotherm indicates the relationship between the solute concentration and adsorption amount in the adsorption equilibrium state at a certain temperature. The shape of an adsorption isotherm depends on the combination of the sorbent and the sorbate. The adsorption isotherm has been widely applied for the evaluation of the adsorption state because its shape represents the interaction between the sorbent and the sorbate. The Langmuir model assumes that (i) the adsorption sites are homogeneous and (ii) the sorbates adsorb at the adsorption sites without interactions with each other. The Langmuir equation is expressed as follows:^40^1
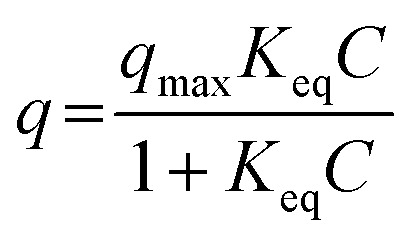
here, *q* represents the amount of the adsorbate at equilibrium (mol g^−1^), *C* represents the concentration of the solute at equilibrium (mol L^−1^), *q*_max_ represents the maximum monolayer adsorption capacity (mol g^−1^), and *K*_eq_ represents the concentration equilibrium constant of adsorption (L mol^−1^). In particular, *q*_max_ and *K*_eq_ are called Langmuir constants; *K*_eq_ indicates the affinity or adsorptivity between the adsorbent and the adsorbate. The Langmuir equation (eqn (1)) can be re-written as follows:2
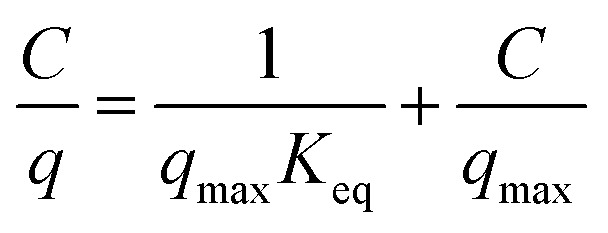


Therefore, the values of *q*_max_ and *K*_eq_ are calculated from the slope and intercept of the linear plot of *C*/*q versus C*.

Gibbs free energy change (Δ*G*) is a significant parameter that is related to the spontaneity of adsorption. Δ*G* depends on the equilibrium constant (*K*), and the relationship between Δ*G* and *K* is as follows:

3 Δ*G* = −*RT* ln  *K*

here, *R* is the gas constant (*R* = 8.314 J mol^−1^ K^−1^) and *T* is the absolute temperature (K). However, the concentration equilibrium constant (*K*_eq_) calculated by the Langmuir equation cannot be substituted into eqn (3) because *K* in eqn (3) needs to be unitless. Thus, *K*_eq_ needs to be converted in the apparent adsorption equilibrium constant, which is explained by kinetics and is unitless.

*K* can be written by using *K*_eq_ as follows:^40^4
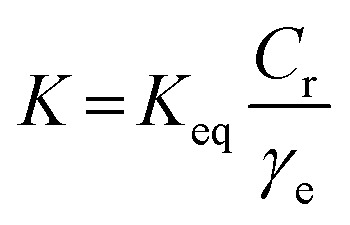
here, *C*_r_, which is the molar concentration of the reference state, is typically 1 mol L^−1^; *γ*_e_ is the activity coefficient (unitless). The magnitude of the activity coefficient depends on the concentration of the ionic adsorbate. Since the concentrations of the acridinium derivatives in this system are weak, *γ*_e_ can be supposed to be 1. Therefore, the apparent equilibrium constant (*K*) is represented as follows:

5 *K* = *K*_eq_*C*_r_

**Fig. 4:**
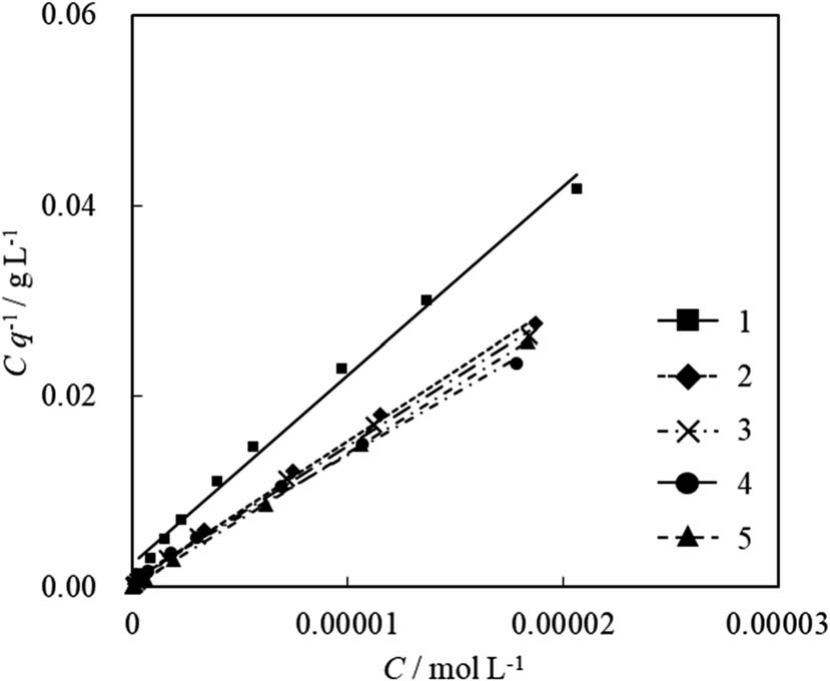
Langmuir isotherm plots for the adsorption of acridinium derivatives on SSA in water at 298.15 K.

Because the absorption spectra of the acridinium derivative-SSA systems were expressed by two-component equilibrium systems of adsorbed and non-adsorbed components, these systems could satisfy the assumption of the Langmuir equation. Hence, we calculated the amount of adsorption from the UV-vis absorption spectra and analyzed the adsorption parameters using Langmuir adsorption isotherms. Fig. 4 shows the Langmuir plots calculated from the absorption spectral changes in Fig. 3. As can be seen, the plots are straight for all acridinium derivatives. The parameters *q*_max_, *K* and Δ*G* calculated from these Langmuir plots are summarized in Table 1.

**Table 1:** Adsorption equilibrium constants and maximum adsorption amounts for acridinium derivatives on SSA

Compound	*K*_eq_/L mol^−1^	Δ*G*/kJ mol^−1^	*q*_max_/% *vs.* CEC
1	8.29 × 10^5^	−33.8	50.6
2	2.01 × 10^6^	−36.0	68.4
3	2.06 × 10^6^	−36.0	71.0
4	1.47 × 10^6^	−35.2	77.4
5	1.05 × 10^7^	−40.0	71.9

In general, it is difficult to analyze the adsorption of mono-cationic molecules on a solid surface by the Langmuir equation because the mono-cationic molecules often aggregate on the solid surface.^41–43^ Δ*G* for adsorption showed negative values for all acridinium derivatives. This result indicates that the adsorption of acridinium derivatives on SSA is an exergonic reaction. Moreover, it was found that the molecular structure influenced the adsorption mechanism because the value of Δ*G* depended on the structure of the substituent. To discuss the adsorption mechanism, a temperature effect experiment was carried out to obtain the values of Δ*H* and Δ*S*.

### 3.3. The temperature effect on adsorption

For adsorption, various interactions such as electrostatic, van der Waals, and hydrophobic interactions can be involved. Δ*G* is composed of Δ*H* and Δ*S*. Among these interactions, it is known that Δ*H* depends on electrostatic and van der Waals interactions and Δ*S* depends on hydrophobic interactions.^32,33^ The relationship among Δ*G*, Δ*H* and Δ*S* is represented as follows:

6 Δ*G* = Δ*H* − *T*Δ*S*

By substituting eqn (3) into eqn (6), van't Hoff equation is obtained:7
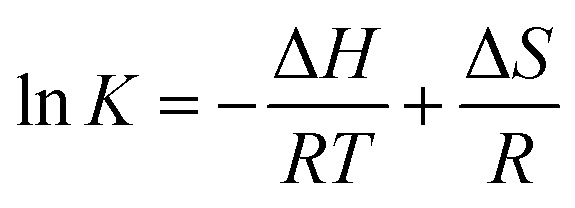


**Fig. 5:**
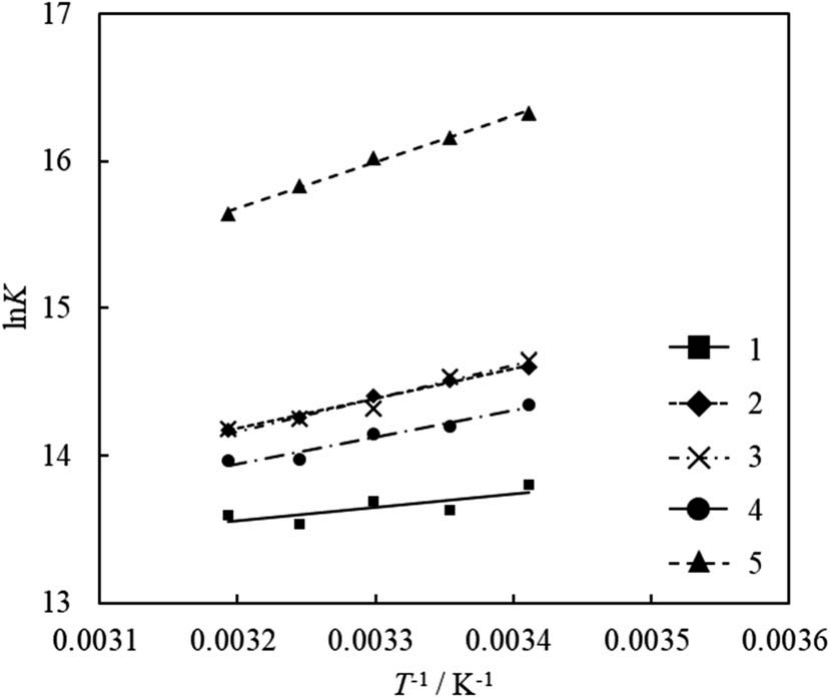
van't Hoff plots for the adsorption of acridinium derivatives on SSA.

The values of Δ*H* and Δ*S* were calculated from the slope and intercept of the linear plot of ln *K versus* 1/*T*. The adsorption equilibrium constants of the acridinium derivatives on SSA at each temperature (293.15, 298.15, 303.15, 308.15, and 313.15 K) were determined by Langmuir adsorption isotherms, and the thermodynamic parameters Δ*H* and Δ*S* were calculated by using these equilibrium constants. Fig. 5 shows the van't Hoff plots and Table 2 shows the values of Δ*H* and Δ*S* for the adsorption of acridinium derivatives on SSA. The UV-vis absorption spectrum and Langmuir adsorption isotherm at each temperature are shown in Fig. S1–S6.†

**Table 2:** Thermodynamic parameters for the adsorption of acridinium derivatives on SSA

Compound	Δ*G*/kJ mol^−1^					Δ*H*/kJ mol^−1^	Δ*S*/kJ mol^−1^ K^−1^	−*T*Δ*S*/kJ mol^−1^ K^−1^
	293 K	298 K	303 K	308 K	313 K			298 K
1	−33.6	−33.8	−34.5	−34.7	−35.4	−7.8	0.088	−25.9
2	−35.6	−36.0	−36.3	−36.5	−36.9	−16.7	0.064	−18.8
3	−35.7	−36.0	−36.1	−36.5	−36.9	−18.6	0.058	−17.4
4	−34.9	−35.2	−35.6	−35.8	−36.4	−14.9	0.068	−20.3
5	−39.8	−40.0	−40.4	−30.5	−40.7	−26.0	0.047	−14.0

**Fig. 6:**
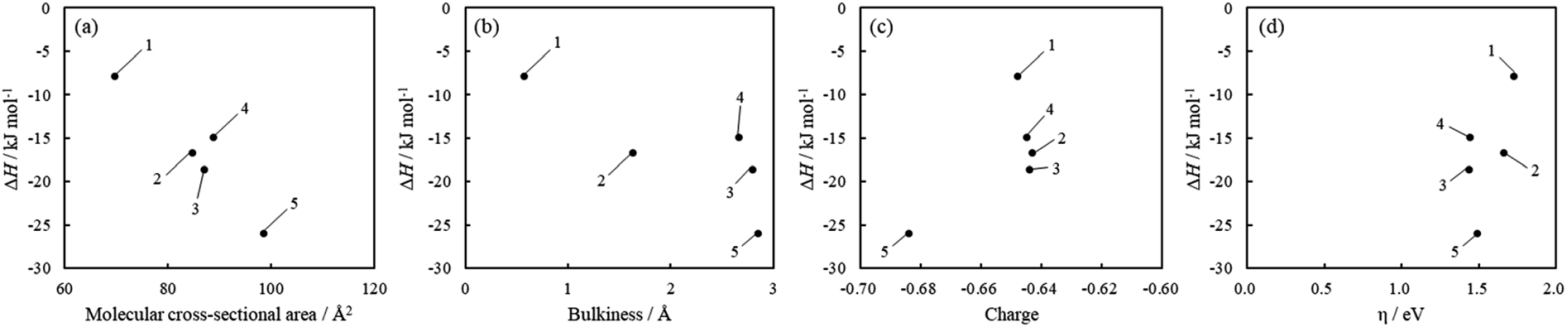
Relationship between Δ*H* and molecular parameters such as (a) molecular cross-sectional area, (b) bulkiness, (c) charge density of N atoms, and (d) chemical hardness. The numbers in the figure are the compound numbers.

The values of Δ*H* were negative for all acridinium derivatives. These results indicate that the adsorption of acridinium derivatives on SSA is an exothermic reaction. To discuss which factors determine the Δ*H* value, the relationship between Δ*H* and molecular parameters such as the molecular cross-sectional area, bulkiness, charge density of N atoms, and chemical hardness (*η*) was examined, as shown in Fig. 6. Pearson *et al.* suggested that chemical hardness can be calculated using electron affinity and ionization energy by the expansion of the Hard and Soft Acids and Bases (HSAB) theory.^44^ Because it is reported that hard acids have higher affinity for hard bases and soft acids have higher affinity for soft bases, chemical hardness is used as a fundamental tool to understand the adsorptivity of the solid surface.^45,46^ Chemical hardness is calculated by eqn (8) using the molecular energy levels of HOMO and LUMO.^44^8
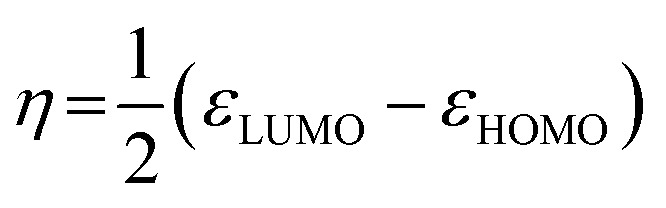


The optimized molecular structures and the estimated energy levels of acridinium derivatives by DFT calculations are shown in Fig. S7 and Table S1.† Δ*H* had a strong correlation with the molecular cross-sectional area (Fig. 6(a)). As the molecular cross-sectional area increased, Δ*H* decreased. Among the interactions, it is expected that van der Waals forces depend on the molecular cross-sectional area.^47^ On the other hand, there was no relationship between Δ*H* and the charge density of N atoms. Judging from these findings, it was suggested that van der Waals forces between the clay surface and adsorbates were predominant for Δ*H* during adsorption. Moreover, since Δ*H* had a strong correlation with the molecular cross-sectional area calculated by assuming that the acridinium derivatives were adsorbed parallelly on SSA, it was suggested that the orientation of the acridinium derivatives on SSA was parallel.

On the other hand, the values of Δ*S* were positive for all acridinium derivatives. In the case of gas-phase adsorption, because the randomness of the gas molecules decreases on the solid surface, the values of Δ*S* are negative in general.^51^ However, various cases have been reported where the Δ*S* of adsorption is positive, especially in solution phase adsorption.^48–50^

**Fig. 7:**
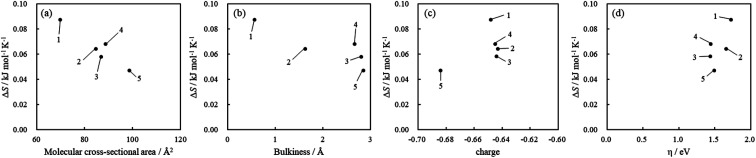
Relationship between Δ*S* and molecular parameters such as (a) molecular cross-sectional area, (b) bulkiness, (c) charge density of N atoms, and (d) chemical hardness. The numbers in the figure are the compound numbers.

For the adsorption of acridinium derivatives on SSA, the positive value of Δ*S* indicated that the randomness in this system increased. Because the water molecules that detached from the solid surface by the adsorption of solute molecules and the iceberg-like water molecules surrounding the solute molecules could obtain entropy, it was suggested that the randomness of the molecules increased. To discuss which factors determined the Δ*S* value, the relationship between Δ*S* and molecular parameters such as the molecular cross-sectional area, bulkiness, charge density of N atoms, and chemical hardness was examined, as shown in Fig. 7. Among them, there was a strong correlation between the molecular cross-sectional area and Δ*S* (Fig. 7(a)). If acridinium derivatives adsorbed parallelly on the clay surface, iceberg-like water would be removed from the surface of the sorbents and sorbates. Therefore, Δ*S* should increase on increasing the molecular cross-sectional area in such a case. However, as the molecular cross-sectional area increased, Δ*S* decreased, as can be seen from Fig. 7. Although the interpretation of the behavior of Δ*S* is not easy, the following one is a possible hypothesis. In the case of acridinium derivatives with small molecular cross-sectional areas such as 1 and 2, they could be adsorbed in an adsorption orientation where the aromatic ring of the acridinium derivatives was parallel to the clay surface. Under such conditions, it is known that Δ*S* increases by adsorption.^3^ In water, a solvent iceberg network should form a hydrogen-bonding network on the surfaces of the complex formed by hydrophobic aromatic rings and clay. The clay surface is thought to be hydrophobic because there are no dangling bonds at oxygen as each oxygen atom is shared by two tetrahedral silicates at the surface of the clay. In fact, it is known that talc, which has very low charge density, is completely hydrophobic. The formation of such icebergs decreases the entropy of the system. To avoid the decrease in entropy, the hydrophobic surfaces tend to face each other in order to decrease their total surface area facing the solvent. Thus, a hydrophobic interaction, which is derived from entropy, would play an important role in water. According to previous literature,^3^ it has been shown that hydrophobic interactions make the parallel orientation of porphyrin stable compared to the tilted orientation. In the case of acridinium derivatives with larger molecular cross-sectional areas and high bulkiness such as 4 and 5, they could be adsorbed in an adsorption orientation where the aromatic ring of the acridinium derivatives was tilted to the clay surface because of their steric effects. In this case, the hydrophobic interactions between the aromatic ring of acridinium derivatives and the clay surface become inefficient. These interpretations are consistent with the tendency shown in Fig. 7(a).

### 3.4. The effect of counter anion on adsorption

**Fig. 8:**
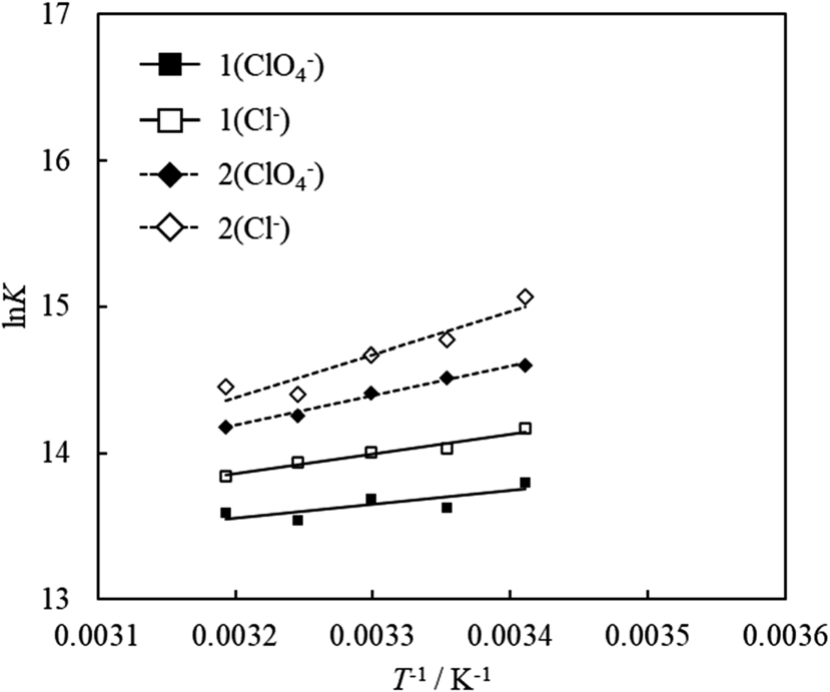
van't Hoff plots for the adsorption of acridinium derivatives (compound 1 (ClO_4_^−^ and Cl^−^) and compound 2 (ClO_4_^−^ and Cl^−^)) on SSA.

To investigate the effect of counter anions on the thermodynamic parameters of adsorption, adsorption experiments were carried out by replacing the counter anions of compound 1 and compound 2 with chloride or perchlorate. Fig. 8 shows the van't Hoff plots for 1 (ClO_4_^−^ and Cl^−^) and 2 (ClO_4_^−^ and Cl^−^). The values of Δ*G*, Δ*H* and Δ*S* obtained from the van't Hoff plots are shown in Table 3. The UV-vis absorption spectrum and Langmuir adsorption isotherm at each temperature are shown in Fig. S8–S10.†

**Table 3:** Thermodynamic parameters for the adsorption of acridinium derivatives (compound 1 (ClO_4_^−^ and Cl^−^) and compound 2 (ClO_4_^−^ and Cl^−^)) on SSA

Compound	Counterion	Δ*G*/kJ mol^−1^					Δ*H*/kJ mol^−1^	Δ*S*/kJ mol^−1^ K^−1^	−*T*Δ*S*/kJ mol^−1^
		293 K	298 K	303 K	308 K	313 K			298 K
1	ClO_4_^−^	−33.6	−33.8	−34.5	−34.7	−35.4	−7.82	0.088	−25.9
1	Cl^−^	−34.5	−34.8	−35.3	−35.7	−36.0	−11.3	0.079	−23.5
2	ClO_4_^−^	−35.6	−36.0	−36.3	−36.5	−36.9	−16.7	0.064	−18.8
2	Cl^−^	−36.7	−36.6	−37.0	−36.9	−37.6	−24.6	0.041	−12.0

The Δ*G* values of compounds 1 and 2 with chloride as the counter anion were smaller than those with perchlorate as the counter anion. This result indicates that adsorption becomes more exergonic by changing the counter anion from perchlorate to chloride. A decrease in Δ*H* and Δ*S* indicates that the contribution of enthalpy has increased. The dissociation of ionic molecules depends on the magnitude of enthalpy. Therefore, the values of enthalpy of dissociation decrease with a decrease in the diameter of the counterion.^52^ In the present study, Δ*H* decreased because the counter anion of the acridinium derivatives was changed from perchlorate to chloride, whose ionic diameter is smaller. This result indicated that the dissociation of the counter anion was reflected in the decrease in Δ*H*. Furthermore, dissociation of the counterion increases the number of water molecules around the dissociated counterion. It was presumed that the contribution of entropy was reduced due to an increase in these water molecules.

## 4. Conclusion

The adsorption behavior of mono-cationic acridinium derivatives on SSA was expressed by two-component equilibrium systems of adsorbed and non-adsorbed components. These results indicated that acridinium derivatives adsorbed on SSA without aggregation. The adsorption equilibrium constants were calculated by analyzing Langmuir adsorption isotherms. The thermodynamic parameters, namely, Δ*G*, Δ*H* and Δ*S* were calculated from van't Hoff plots by using adsorption equilibrium constants at each temperature. For all acridinium derivatives, Δ*G* (at 298.15 K), Δ*H* (at 298.15 K) and Δ*S* were calculated to be in the range from −33.8 to −40.0 kJ mol^−1^, −7.82 to −26.0 kJ mol^−1^ and 0.047 to 0.088 kJ mol^−1^ K^−1^, respectively. It was found that the adsorption of the acridinium derivatives on SSA was an exothermic reaction because Δ*H* showed negative values for all derivatives. It was found that both van der Waals and hydrophobic interactions contributed to the adsorption of mono-cationic acridinium derivatives on SSA because Δ*H* showed negative values and Δ*S* showed positive values. These thermodynamic parameters had good correlations with the molecular cross-sectional areas of acridinium derivatives as sorbates. Moreover, the Δ*G* and Δ*H* of compounds 1 and 2 with chloride as the counter anion were smaller than those with perchlorate as the counter anion. The decrease in Δ*G* suggested that the acridinium derivatives with chloride as the counter anion, a highly dissociable counter anion, had higher adsorptivity than the acridinium derivatives with perchlorate as the counter anion. The decrease in Δ*H* suggested that the contribution of enthalpy increased when a higher dissociable ion, such as a chloride ion, was the counter anion of the sorbate.

## Conflicts of interest

There are no conflicts to declare.

## Supplementary Material

RA-010-D0RA03158E-s001
