# Supplementary material for: Thermodynamic study of the adsorption of acridinium derivatives on the clay surface
Source: RSC Adv. 2020 Jun 4;10(36):21360–8. doi: 10.1039/d0ra03158e (PMC9054366; doi:10.1039/d0ra03158e)
Supplement: RA-010-D0RA03158E-s001 [file RA-010-D0RA03158E-s001.pdf]

Electronic Supplementary Material (ESI) for RSC Advances.
This journal is © The Royal Society of Chemistry 2020

[Supporting information]

Thermodynamic study for Adsorption of Acridinium Derivatives on the Clay Surface

*Yuma Yoshida,[1] Tetsuya Shimada,[1,2] Tamao Ishida,[1,2,3] Shinsuke Takagi[1,2] **

[1]Department of Applied Chemistry, Graduate Course of Urban Environmental Sciences, Tokyo Metropolitan University, Minami-ohsawa 1-1, Hachiohji, Tokyo 192-0397 Japan

[2]Research Center for Hydrogen Energy-based Society (ReHES), Tokyo Metropolitan University, 1-1 Minami-ohsawa 1-1, Hachiohji, Tokyo 192-0397 Japan

[3]Research Center for Gold Chemistry, Tokyo Metropolitan University, Minami-ohsawa 1-1, Hachiohji, Tokyo 192-0397 Japan

takagi-shinsuke@tmu.ac.jp

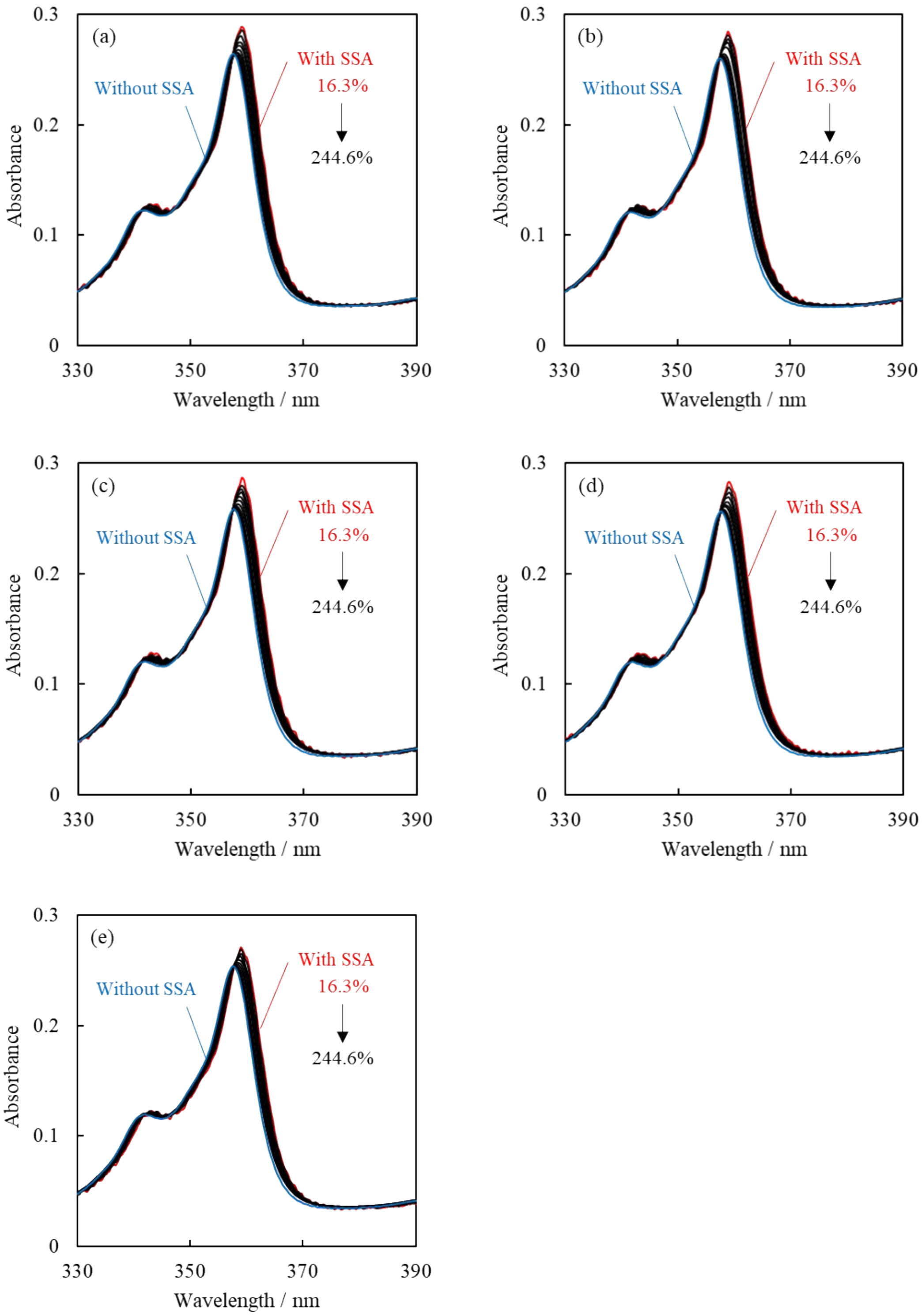

Figure S1. UV-vis adsorption spectra of compound **1** (perchlorate) with SSA and without SSA in water. The loading levels of compound 1 were 16.3, 24.5, 32.6, 40.8, 48.9, 65.2, 81.5, 122.3, 163.0, 244.6% vs. CEC: (a)273.15 K, (b)278.15 K, (c)283.15 K, (d)288.15 K, (e)293.15 K.

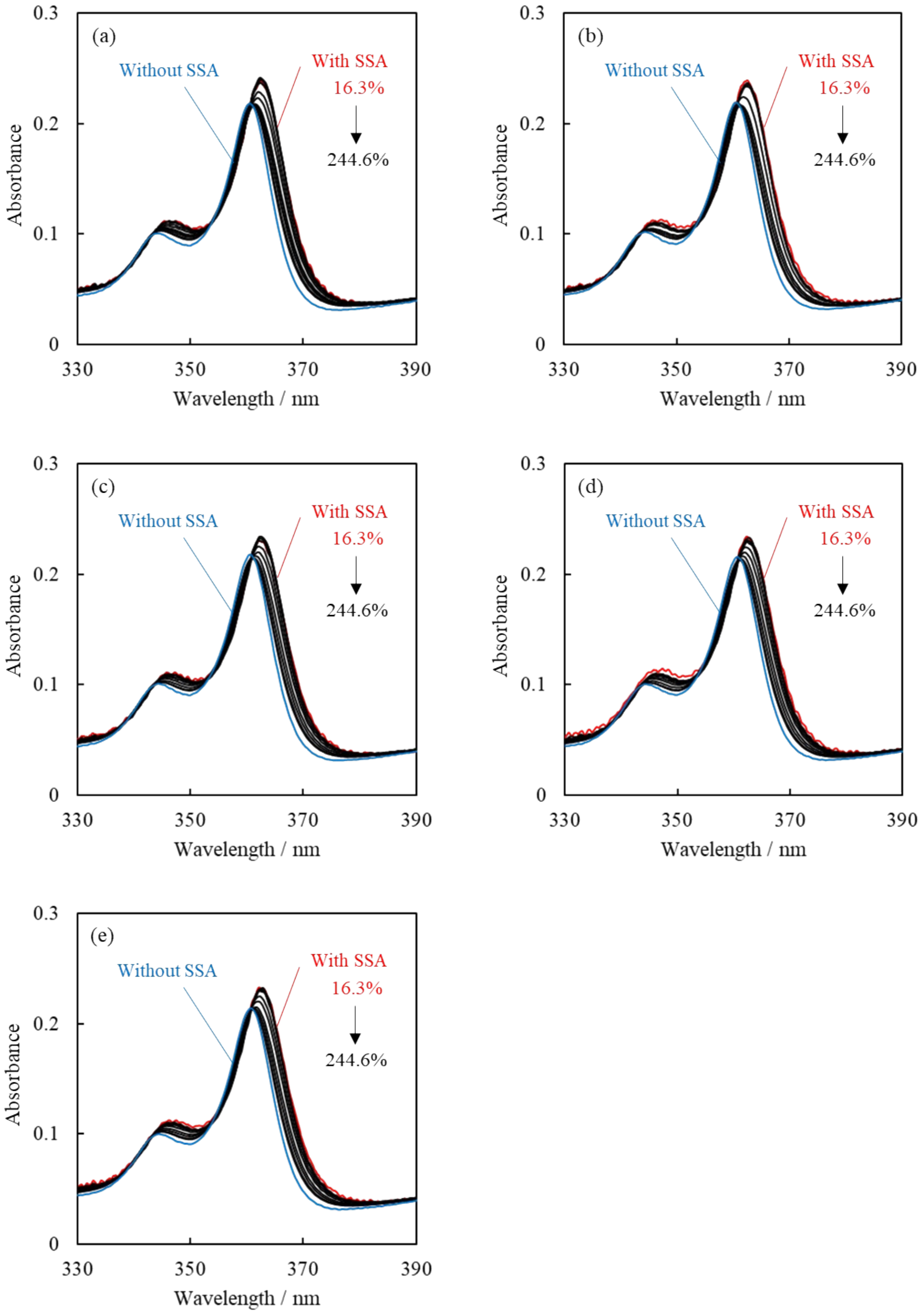

Figure S2. UV-vis adsorption spectra of compound **2** (perchlorate) with SSA and without SSA in water. The loading levels of compound 2 were 16.3, 24.5, 32.6, 40.8, 48.9, 65.2, 81.5, 122.3, 163.0, 244.6% vs. CEC: (a)273.15 K, (b)278.15 K, (c)283.15 K, (d)288.15 K, (e)293.15 K.

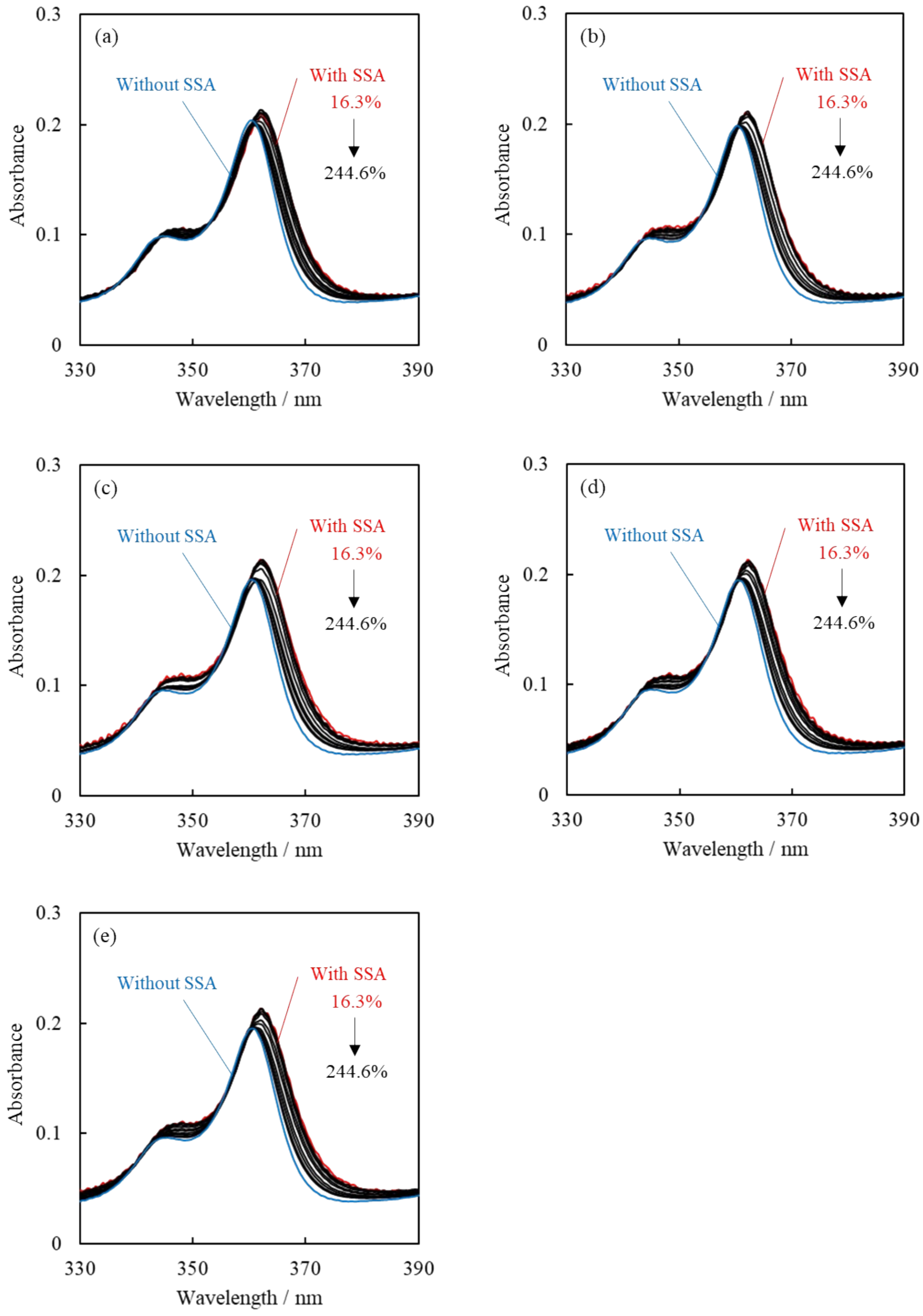

Figure S3. UV-vis adsorption spectra of compound **3** (perchlorate) with SSA and without SSA in water. The loading levels of compound 3 were 16.3, 24.5, 32.6, 40.8, 48.9, 65.2, 81.5, 122.3, 163.0, 244.6% vs. CEC: (a)273.15 K, (b)278.15 K, (c)283.15 K, (d)288.15 K, (e)293.15 K.

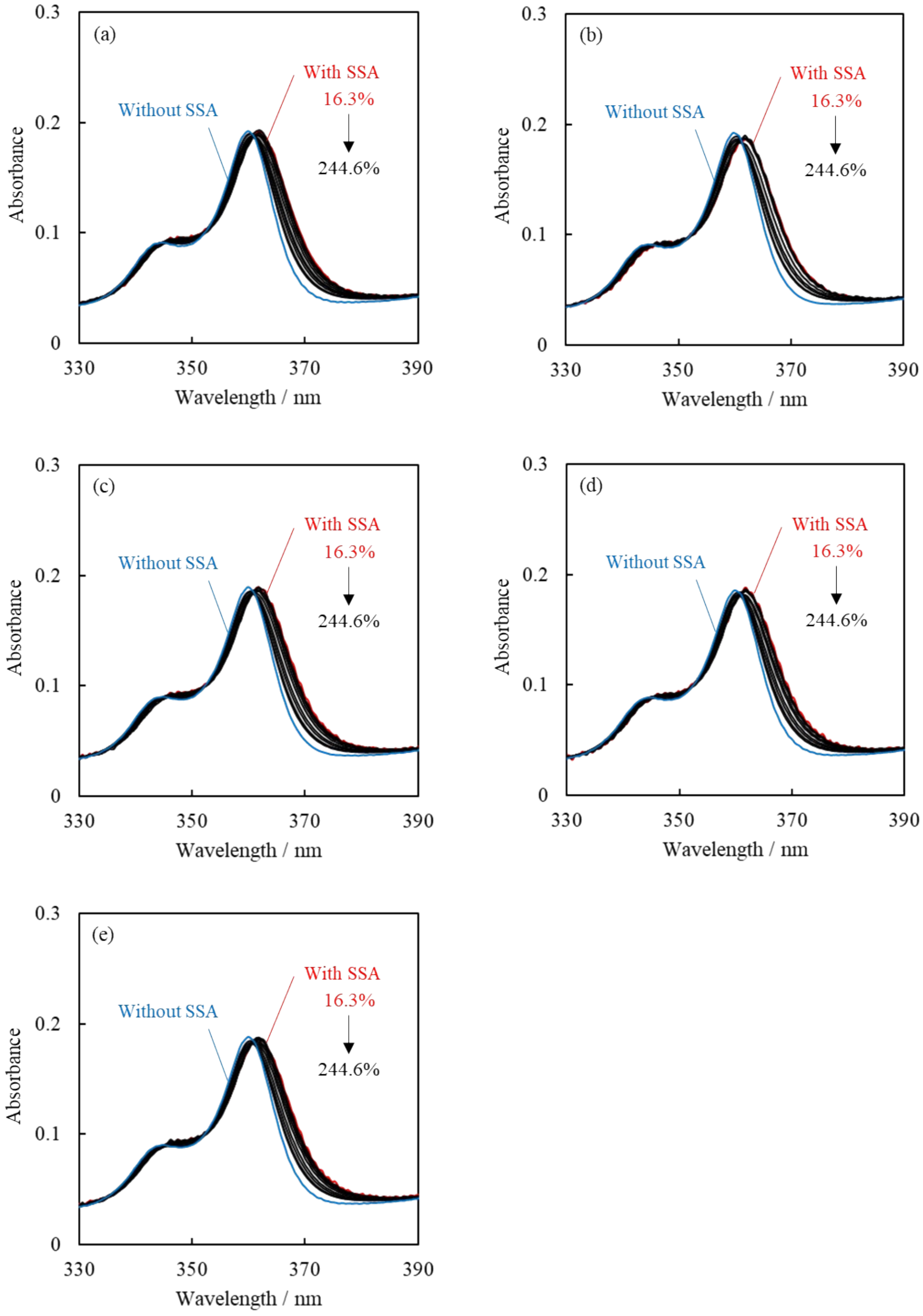

Figure S4. UV-vis adsorption spectra of compound **4** (perchlorate) with SSA and without SSA in water. The loading levels of compound 4 were 16.3, 24.5, 32.6, 40.8, 48.9, 65.2, 81.5, 122.3, 163.0, 244.6% vs. CEC: (a)273.15 K, (b)278.15 K, (c)283.15 K, (d)288.15 K, (e)293.15 K.

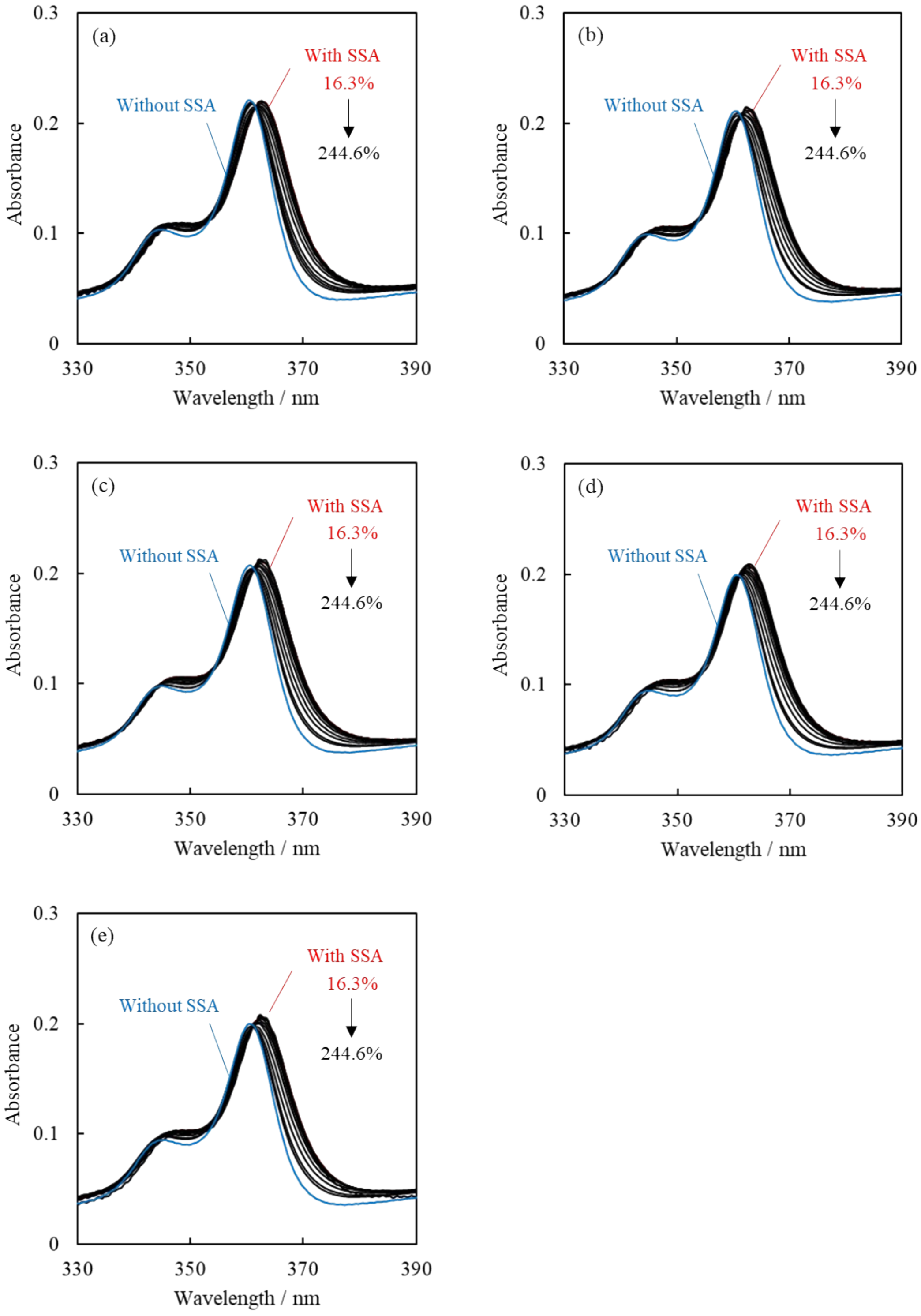

Figure S5. UV-vis adsorption spectra of compound **5** (perchlorate) with SSA and without SSA in water. The loading levels of compound 5 were 16.3, 24.5, 48.9, 57.1, 65.2, 73.4, 81.5, 97.8, 122.3, 163.0, 244.6% vs. CEC: (a)273.15 K, (b)278.15 K, (c)283.15 K, (d)288.15 K, (e)293.15 K.

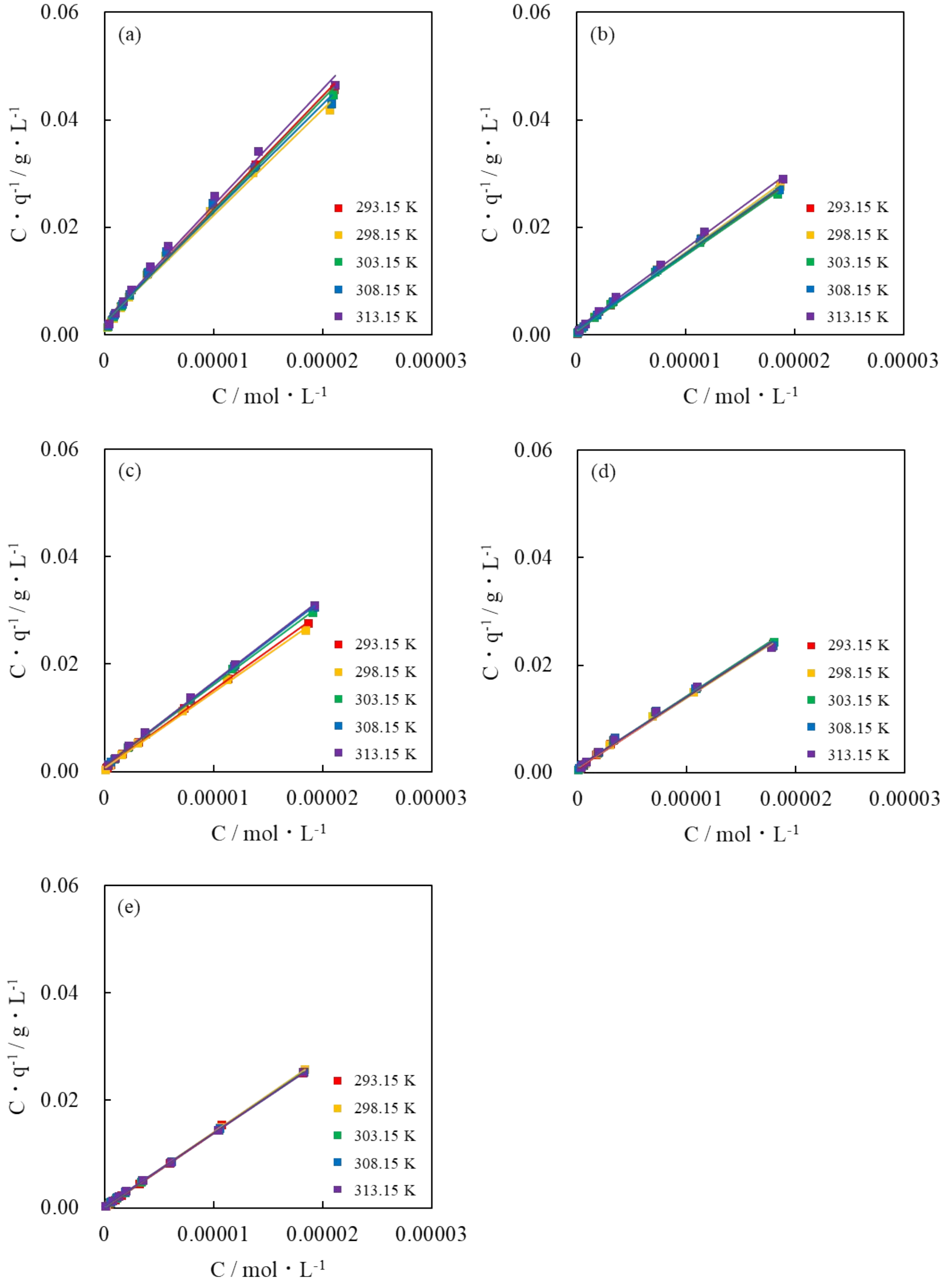

Figure S6. Langmuir isotherm analysis for the adsorption of acridinium derivatives (perchlorate) on SSA at each temperature: (a) compound **1**, (b) compound **2**, (c) compound **3**, (d) compound **4**, (e) compound **5**.

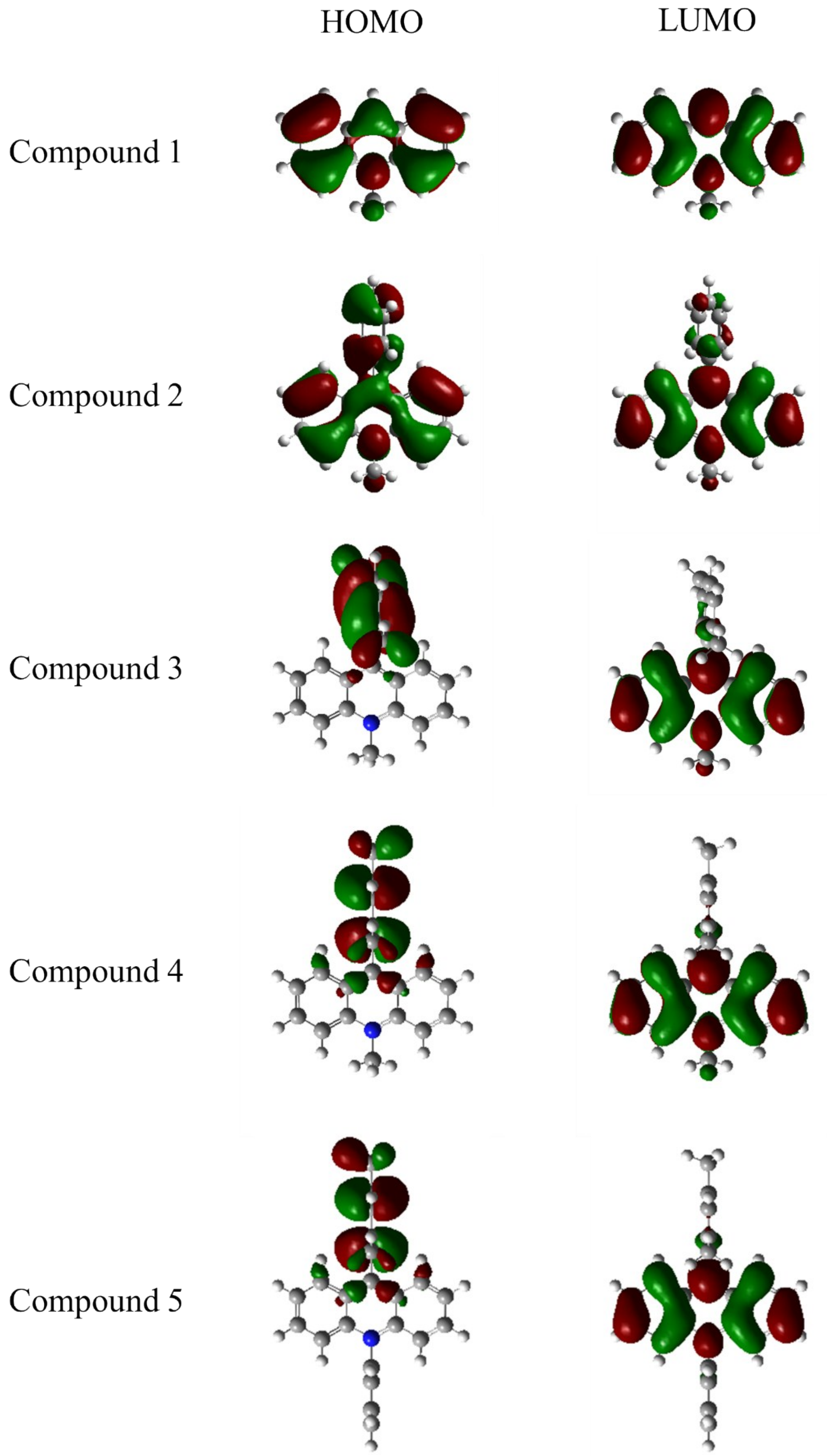

Figure S7. Optimized molecular structure of acridinium derivatives (compound 1–5) obtained from DFT calculations performed at B3LYP/6-31G* level using Gaussian09 package.

Table S1. Estimated energy levels of HOMO and LUMO for acridinium derivatives (compound 1–5) and chemical hardness

Compound	ε_{HOMO} / eV	ε_{LUMO} / eV	η / eV
1	-10.119	-6.671	1.724
2	-9.678	-6.359	1.659
3	-9.201	-6.311	1.445
4	-9.095	-6.110	1.493
5	-9.196	-6.322	1.437

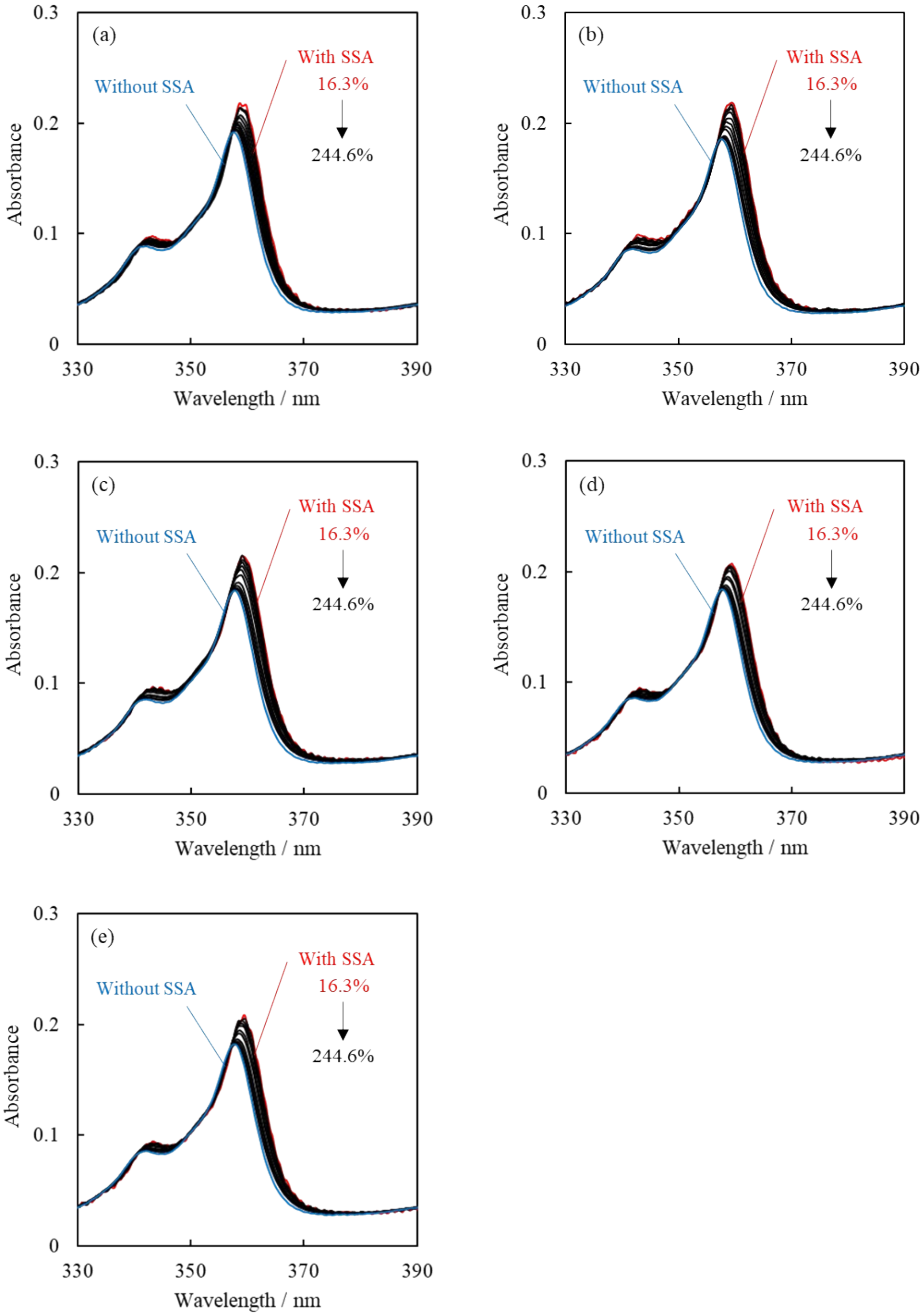

Figure S8. UV-vis adsorption spectra of compound 1 (chloride) with SSA and without SSA in water. The loading levels of compound 1 were 16.3, 24.5, 32.6, 40.8, 48.9, 65.2, 81.5, 122.3, 163.0, 244.6% vs. CEC: (a)273.15 K, (b)278.15 K, (c)283.15 K, (d)288.15 K, (e)293.15 K.

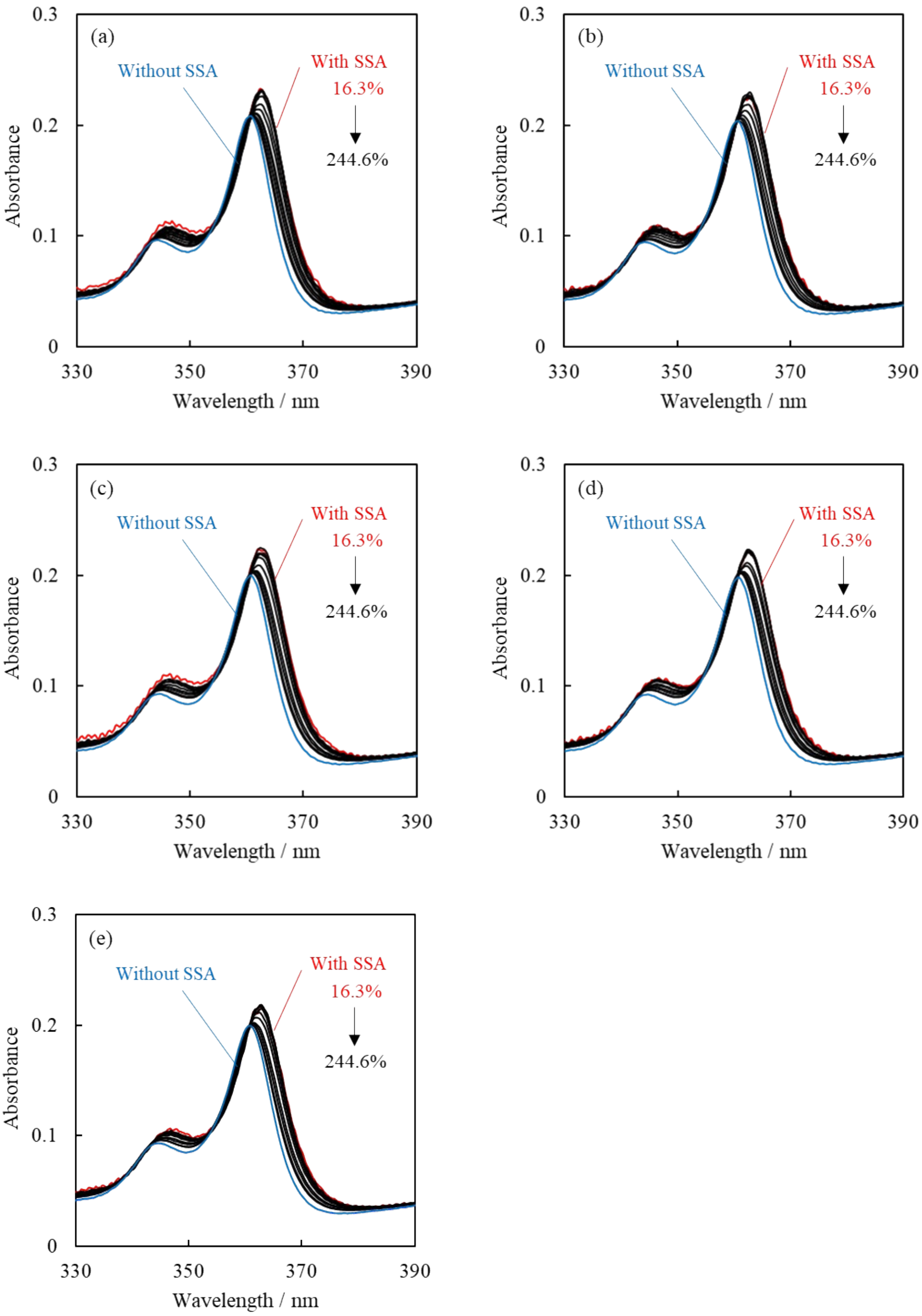

Figure S9. UV-vis adsorption spectra of compound 2 (chloride) with SSA and without SSA in water. The loading levels of compound 1 were 16.3, 24.5, 32.6, 40.8, 48.9, 65.2, 81.5, 122.3, 163.0, 244.6% vs. CEC: (a)273.15 K, (b)278.15 K, (c)283.15 K, (d)288.15 K, (e)293.15 K.

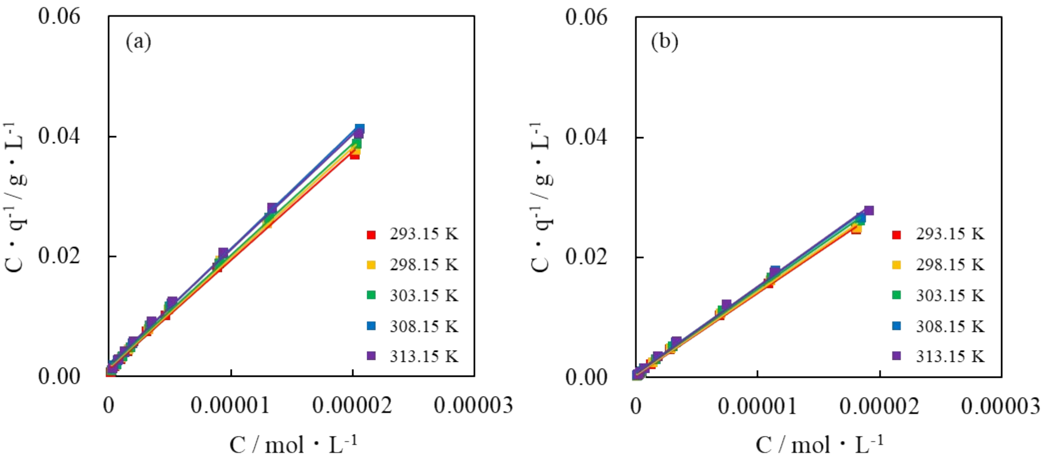

Figure S10. Langmuir isotherm analysis for the adsorption of Acridinium derivatives (chloride) on SSA at each temperature: (a) compound 1, (b) compound 2.
